# Annual Dynamics of Endogenous Hormones Reveal the Mechanism of Off-Season Flowering in Macadamia

**DOI:** 10.3390/plants14172637

**Published:** 2025-08-24

**Authors:** Ya Ning, Yuchun Chen, Xiyong He, Tingmei Yang, Hai Yue

**Affiliations:** 1 Yunnan Institute of Tropical Crops, Xishuangbanna 666100, China; 2 Macadami Agricultural Engineering Research Center of Yunnan Province, Xishuangbanna 666100, China

**Keywords:** *Macadamia*, off-season flowering, endogenous hormones, hormone ratio, floral induction, phenological regulation

## Abstract

Off-season flowering of Macadamia integrifolia has been observed in certain high-altitude regions; however, the endogenous hormonal mechanisms underlying this phenomenon remain unclear. In this study, the annual dynamics of four key endogenous phytohormones (ZT, GA_3_, IAA, and ABA) were quantified in the leaves and branches of trees from both normal and off-season flowering sites using high-performance liquid chromatography (HPLC). Hormonal ratios and correlation network analyses were further performed to investigate their roles in flowering regulation. Floral bud differentiation at the off-season site occurred approximately 1 to 2 months earlier than at the normal site. This advancement was associated with sustained low levels of GA_3_ (below 100 μg·g^−1^ FW), while ZT and ABA levels peaked in September at 108.66 μg·g^−1^ FW and 24.25 μg·g^−1^ FW, respectively. The ratios of ABA to GA_3_, ABA to IAA, and ZT to GA_3_ increased significantly between July and September, indicating the early establishment of a hormonal environment favorable for floral induction. Correlation analysis revealed that IAA, GA_3_, and ZT formed a synergistic module promoting flowering, whereas ABA functioned as an antagonistic regulator. These findings provide insight into the hormonal regulation of off-season flowering in macadamia and offer a theoretical basis for precision flowering control in high-altitude cultivation systems.

## 1. Introduction

**Figure 1:**
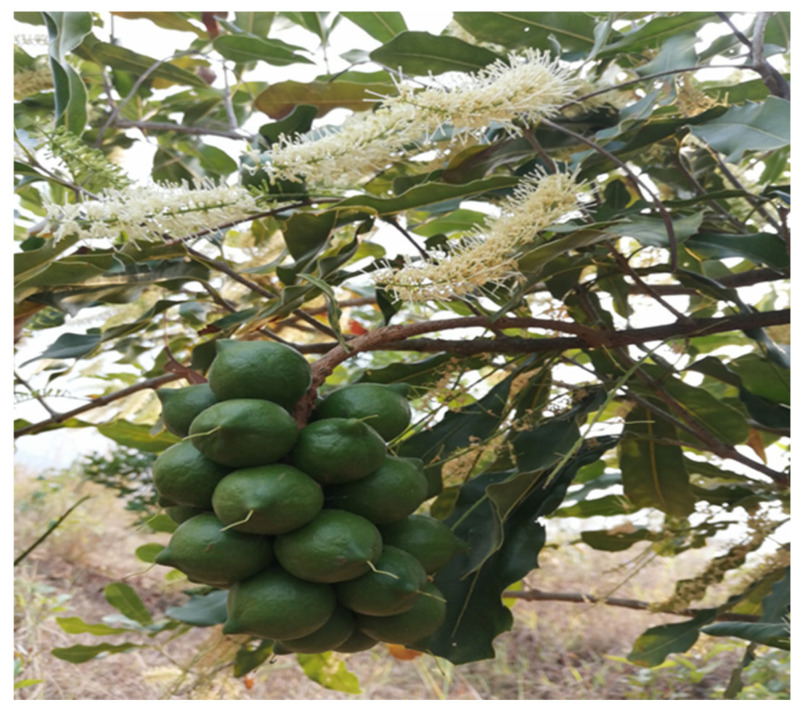
Off-season flowering in macadamia, with the co-occurrence of developing. Fruits and floral inflorescences on the same tree.

Macadamia (*Macadamia* spp.) is an evergreen tree species native to the subtropical rainforests of southeastern Queensland and northeastern New South Wales, Australia [1,2]. Macadamia nuts are rich in unsaturated fatty acids, proteins, amino acids, and various vitamins, making them highly valuable to both the food processing and cosmetics industries [3]. In recent years, China has emerged as the world’s leading producer of macadamia, with Yunnan Province alone accounting for nearly 50% of the global cultivation area [4]. However, the rapid expansion of macadamia plantations has been accompanied by an increasing prevalence of off-season flowering in certain production regions. This atypical phenological pattern disrupts the synchrony of pollination and fertilization, leading to reduced fruit set, compromised fruit quality, and yield fluctuations ranging from 20% to 30% [5]. Such disturbances not only threaten the stability of the supply chain but also pose ecological risks by misaligning macadamia phenology with pollinator activity [6]. Figure 1 shows the occurrence of off-season flowering in macadamia, with the co-occurrence of developing fruits and floral inflorescences on the same tree. Furthermore, off-season flowering results in asynchronous fruit development within individual trees, thereby complicating harvesting operations and elevating labor costs. When fruits are harvested in a single batch, overall quality often deteriorates, further constraining the sustainable development of the macadamia industry [7].

The flowering process in plants is regulated by a complex interplay of internal and external factors, including photoperiod, temperature, water availability, nutrient status, and endogenous phytohormones [8]. Among these, endogenous hormones function as central integrators, translating environmental cues into developmental responses. They play essential roles in floral bud differentiation, dormancy release, and the initiation of flowering [9]. The major classes of phytohormones involved in these processes include gibberellins (Gas), indole-3-acetic acid (IAA), cytokinins (CTKs), and abscisic acid (ABA) [10]. Gibberellins are particularly well known for their involvement in multiple regulatory pathways that promote the transition from vegetative to reproductive growth. However, in macadamia, Gas may inhibit floral bud differentiation [11,12], underscoring the species-specific complexity and context-dependent nature of hormonal regulation.

IAA is essential for the initiation of floral primordia, with IAA deficiency known to impair flower formation. CTKs regulate floral meristem activity by modulating cell division and differentiation [12,13]. ABA, widely recognized for its role in abiotic stress responses, is generally considered a floral repressor. Elevated ABA levels are frequently associated with delayed floral bud development and reduced flowering potential [14]. Recent evidence suggests that flowering regulation by endogenous hormones is not solely determined by the absolute concentrations of individual hormones, but rather by the dynamic balance and interactions among them. Hormonal ratios, such as ZT/GA_3_, ZT/IAA, ABA/IAA, and ABA/GA_3_, have been shown to play critical roles in determining floral developmental outcomes [15,16]. Higher ratios of ZT/GA_3_, ZT/IAA, ABA/IAA, and ABA/GA_3_ are typically associated with the maintenance of vegetative bud states, whereas lower values are more conducive to the transition toward reproductive development [17]. Therefore, elucidating the hormonal regulatory mechanisms underlying off-season flowering in macadamia is essential for understanding its phenological plasticity and for developing effective strategies to mitigate production instability. To address this, the present study investigates the hormonal signaling pathways associated with off-season flowering in high-altitude macadamia by comparing hormone levels and ratios in contrasting phenological regions over a 12-month period, aiming to provide insights for flowering regulation strategies.

## 2. Results

### 2.1. Phenological Observation of Macadamia

A systematic observation and comparison of the annual phenological stages were conducted at the two study sites. The results revealed significant differences between the MH and JH sites in the timing of key developmental events, including floral bud differentiation, flowering, fruit development, and maturation (Table 1). At the MH site, floral bud differentiation primarily occurred from August to September. Initial flowering was observed in October and November, with peak flowering extending into January of the following year. Fruit entered the enlargement stage from February to April, followed by oil accumulation from May to July, and reached near maturity by August. In contrast, all corresponding phenophases at the JH site were delayed by approximately 1 to 2 months. Floral bud differentiation occurred from October to December, initial flowering shifted to January–February of the following year, peak flowering occurred in March, fruit enlargement and oil accumulation took place from April–May to June–August, respectively, and maturation and harvest began in September.

### 2.2. Dynamics of Endogenous Hormones in Macadamia

#### 2.2.1. Dynamic Changes of Endogenous Hormones in Leaves

**Figure 2:**
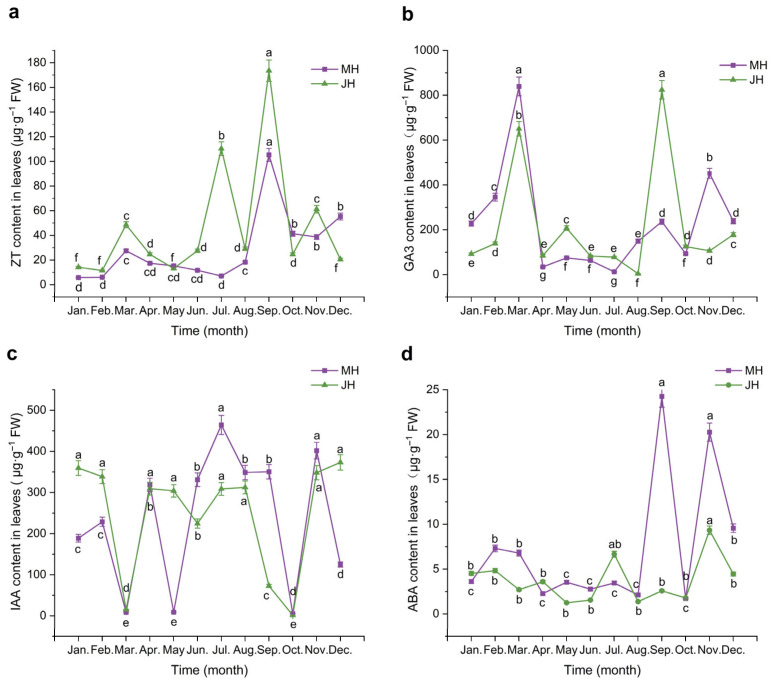
Dynamic changes in endogenous hormone contents in *macadamia* leaves at MH (purple line) and JH (green line) sites. (**a**) Zeatin (ZT); (**b**) Gibberellic acid (GA_3_); (**c**) Indole-3-acetic acid (IAA); (**d**) Abscisic acid (ABA). Error bars represent the standard error (n = 5). Different lowercase letters indicate significant differences (*p* < 0.05) among months within the same site based on one-way ANOVA followed by the LSD test.

The endogenous hormone contents in macadamia leaves were measured monthly from January to December, revealing the dynamic changes over the course of the year (Figure 2).

Zeatin (ZT) content in macadamia leaves exhibited pronounced seasonal variation across both study sites. At the MH site, ZT levels remained consistently low and stable from January to August, followed by a sharp increase beginning in late August and peaking in September at 105.28 µg·g^−1^ FW. A marked decline occurred in October, with a slight rebound from November to December, maintaining a moderately low level overall. At the JH site, ZT content was also low from January to June, then increased sharply in July to 110.32 µg·g^−1^ FW. After a minor dip in August, ZT levels rose again in September, reaching the annual maximum of 123.56 µg·g^−1^ FW, followed by a notable decline in October and a modest recovery in November and December.

Gibberellic acid (GA_3_) concentrations exhibited distinct seasonal dynamics between the two sites. At the MH site, GA_3_ content increased steadily from January to March, reaching an early-year peak in March (839.08 µg·g^−1^ FW). This was followed by a sharp decline in April, and relatively low and stable levels were maintained from April through July. A secondary rise occurred in August and September, followed by a decline in October, a slight rebound in November, and a modest decrease in December. At the JH site, the early-year trend (January to March) was similar, with a peak in March. However, GA_3_ levels rose significantly again in May before gradually decreasing to the annual minimum in August. A strong rebound was observed in September, resulting in the highest yearly peak at 824.31 µg·g^−1^ FW, after which levels declined rapidly to early-year values.

Indole-3-acetic acid (IAA) levels at the MH site exhibited a “three peaks and three troughs” pattern over the course of the year. Concentrations were moderately high from January to February, followed by a sharp decline in March, reaching the first trough. A rapid rebound occurred in April, followed by a slight decline in May, a rise in June, and the highest annual peak in July (464.27 µg·g^−1^ FW). IAA levels remained relatively elevated through September, then dropped sharply in October to the lowest point of the year (5.25 µg·g^−1^ FW). A significant increase was observed in November, followed by a minor decline in December. At the JH site, IAA content was also relatively high in January and February but decreased abruptly to 15.40 µg·g^−1^ FW in March. From April to August, levels remained relatively stable and elevated, followed by a sharp decline from September to October, reaching a second trough in October (1.35 µg·g^−1^ FW). IAA content then rose sharply in November and stabilized at a moderate level in December.

Abscisic acid (ABA) concentrations remained relatively low throughout the observation period compared with other hormones. At the MH site, two distinct peaks were observed in September and November, with maximum values of 24.25 and 20.27 µg·g^−1^ FW, respectively. During the remaining months, ABA content was consistently low and exhibited minimal fluctuations. At the JH site, ABA variation was even more constrained, with peak concentrations lower than those at the MH site. The highest annual concentration occurred in November (9.34 µg·g^−1^ FW), and levels remained consistently lower than those at MH during most of the year.

#### 2.2.2. Dynamic Changes of Endogenous Hormones in Branches

**Figure 3:**
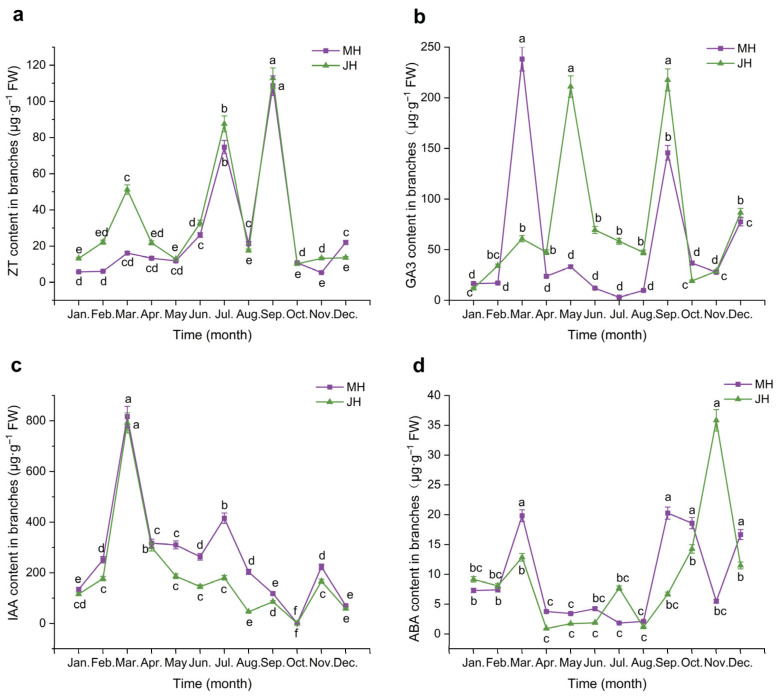
Dynamic changes in endogenous hormone contents in *macadamia* branches at MH (purple line) and JH (green line) sites. (**a**) Zeatin (ZT); (**b**) Gibberellic acid (GA_3_); (**c**) Indole-3-acetic acid (IAA); (**d**) Abscisic acid (ABA). Error bars represent the standard error (n = 5). Different lowercase letters indicate significant differences (*p* < 0.05) among months within the same site, as determined by one-way ANOVA followed by the LSD test.

Annual variations in the concentrations of four endogenous hormones, including zeatin (ZT), gibberellic acid (GA_3_), indole-3-acetic acid (IAA), and abscisic acid (ABA), in macadamia branches are illustrated in Figure 3, and the corresponding temporal patterns are described below.

Zeatin (ZT) content in macadamia branches exhibited similar bimodal seasonal trends at both sites, though with notable temporal variations. At the MH site, ZT levels remained low from January to May, followed by a sharp increase beginning in June, reaching the first peak in July (74.70 μg·g^−1^ FW). After a brief decline, levels rose again to the annual maximum in September (108.66 μg·g^−1^ FW) and then declined rapidly from October to December. The JH site displayed a comparable pattern with two prominent peaks also in July and September. However, JH exhibited a small additional peak in March (51.26 μg·g^−1^ FW), which was absent at the MH site. Moreover, the increase in ZT content during June was less marked at JH than at MH, indicating potential differences in ZT regulatory timing between the two sites.

Gibberellic acid (GA_3_) levels in branches demonstrated more pronounced pulse-like fluctuations at the MH site than at JH. At MH, GA_3_ content remained low in January and February, followed by a sharp surge in March that resulted in the annual maximum (238.22 μg·g^−1^ FW). Levels then declined rapidly and remained low from April to August, with a minimum in July (3.02 μg·g^−1^ FW). A second peak was observed in September (145.62 μg·g^−1^ FW), followed by a decline in October and a minor rebound in November and December. In contrast, GA_3_ levels at JH fluctuated more smoothly. After low levels in January and February, a modest rise in March was followed by the annual peak in May (211.01 μg·g^−1^ FW). Levels then declined until August, increased again in September (217.62 μg·g^−1^ FW), and dropped in October, stabilizing over the final two months. Notably, the first peak at JH occurred approximately two months later than at MH.

Indole-3-acetic acid (IAA) in branches displayed a distinct seasonal rhythm, with similar trends at both sites. At MH, IAA content was low in January but rose sharply in February, reaching the annual maximum in March at 816.57 μg·g^−1^ FW, which represented the highest annual value. Levels then declined rapidly and stabilized from April to June. A secondary moderate peak occurred in July (415.47 μg·g^−1^ FW), followed by a decline to the lowest annual value in October (0.75 μg·g^−1^ FW). Slight rebounds occurred in November, and a small decrease followed in December. At the JH site, the pattern was comparable but with slightly lower amplitudes. IAA content gradually increased from January to March (peak: 791.78 μg·g^−1^ FW), then declined sharply through June. A minor rise was observed in July, followed by a steady decrease until October (minimum: 0.35 μg·g^−1^ FW), with a moderate rebound in November and slight decline in December.

Abscisic acid (ABA) content in branches varied seasonally and showed contrasting peak timings between the two sites. At the MH site, ABA levels were low during January and February, then rose sharply in March, forming the first major peak. Levels dropped substantially from April to July, with the lowest value recorded in July. A notable increase in August occurred, culminating in a second peak in September, followed by a slight drop in October and another moderate increase in December, forming the third-highest annual value. At the JH site, the pattern was somewhat different: ABA levels also remained low from January to April (minimum in April), then began rising in July and rose steadily, reaching the annual maximum in November (35.83 μg·g^−1^ FW), followed by a sharp decline in December. Overall, the timing of peak ABA levels differed between sites, with the MH site peaking in September and the JH site in November, and an opposing trend observed during July.

### 2.3. Dynamic Changes in the Endogenous Hormone Balance Ratios in Macadamia

**Figure 4:**
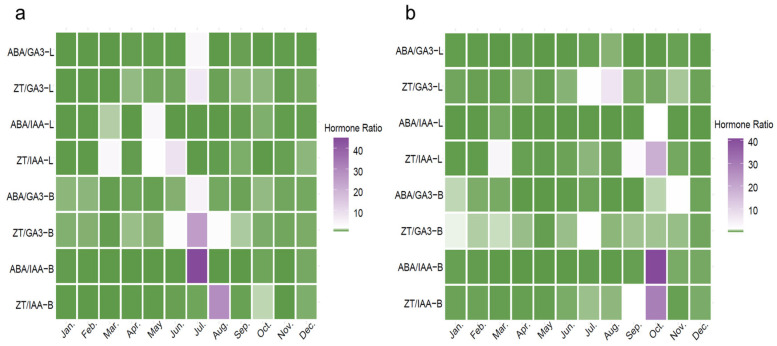
Dynamic changes in the balance ratios of endogenous hormones in *macadamia* at MH (**a**) and JH (**b**) sites. Heatmaps present monthly variations in four hormone ratios (ABA/GA_3_, ZT/GA_3_, ABA/IAA, and ZT/IAA) in leaves (L) and branches (B) from January to December. Color intensity indicates the relative magnitude of the ratios (unitless), as shown by the scale bar.

To further evaluate the regulatory role of hormonal interactions, a heat map was generated to illustrate the monthly changes in four key hormone ratios: ABA/GA_3_, ZT/GA_3_, ABA/IAA, and ZT/IAA. The color gradient ranged from green to purplish-red, representing a transition from low to high ratio values. The results (Figure 4) revealed significant spatiotemporal variation in hormonal balance across different sites and tissue types.

At the MH site (Figure 4a), hormone ratios in both leaves (L) and branches (B) exhibited distinct seasonal patterns. In branches, the ABA/IAA and ZT/IAA ratios showed intensified coloration in July and August, indicating marked increases and the highest annual values during this period. Additionally, the ZT/GA_3_ ratio also rose significantly in July. In leaves, ZT/IAA and ABA/IAA showed moderate increases between April and June, whereas ABA/GA_3_ and ZT/GA_3_ remained relatively stable throughout the year, with slight elevations from June to July. Overall, hormone ratio peaks in branches were concentrated in midsummer, while fluctuations in leaves were more moderate and sustained.

At the JH site (Figure 4b), the seasonal dynamics of hormone ratios differed notably from those observed at MH. In branches, the ABA/IAA and ZT/IAA ratios increased sharply in October, reaching their highest annual levels. The ZT/GA_3_ and ABA/GA_3_ ratios exhibited only minor fluctuations throughout the year, showing relatively uniform coloration with slight increases in July and November. These patterns suggest a generally stable hormonal balance in branches, without pronounced high-ratio periods. In leaves, variations were minimal; the ZT/IAA ratio showed a slight increase and ABA/IAA a marginal rise in October, while changes in other months were negligible. The ZT/GA_3_ and ABA/GA_3_ ratios in leaves were characterized by lighter coloration in July and August, and ABA/GA_3_ remained relatively stable throughout the year, indicating limited seasonal variation.

### 2.4. Correlation Analysis of Endogenous Hormones and Their Ratios in Macadamia

**Figure 5:**
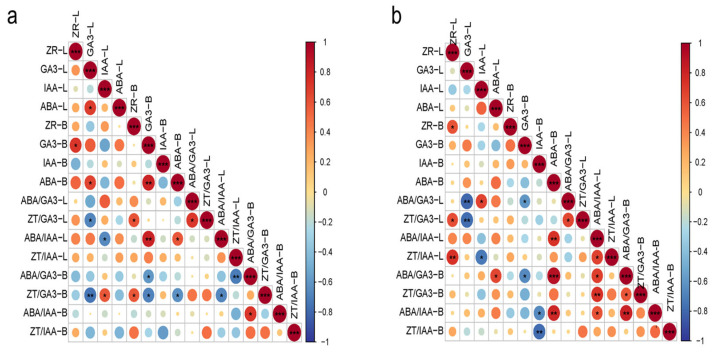
Correlation analysis of endogenous hormones and their ratios in macadamia at MH (**a**) and JH (**b**) sites. The size and color intensity of the circles indicate the strength of the correlation. Red circles represent positive correlations, and blue circles represent negative correlations. An asterisk (*) indicates statistically significant correlations (*p* < 0.05), two asterisks (**) indicate highly significant correlations (*p* < 0.01), and three asterisks (***) indicate very highly significant correlations (*p* < 0.001).

To elucidate the interrelationships among endogenous hormones and their ratios in macadamia, Spearman correlation analysis was performed on hormone concentrations from leaves and branches at the two sampling sites (MH and JH) (Figure 5). The results revealed distinct differences in correlation patterns between the two sites.

At the MH site (Figure 5a), a significant positive correlation was observed between ABA and GA_3_ in leaves. GA_3_ levels in branches showed a significant positive correlation with leaf ZT, and GA_3_ also displayed positive correlations with ABA in both leaves and branches. The leaf ZT/GA_3_ ratio was negatively correlated with leaf GA_3_ but positively associated with branch ZT. The leaf ABA/IAA ratio showed a negative correlation with leaf IAA, while exhibiting a strong positive correlation with branch GA_3_ and a significant positive correlation with branch ABA. The branch ABA/GA_3_ ratio was negatively correlated with branch GA_3_ and strongly negatively correlated with the leaf ZT/IAA ratio. The branch ZT/GA_3_ ratio was negatively associated with leaf GA_3_, branch GA_3_, branch ABA, and the leaf ABA/IAA ratio, while showing a positive correlation with leaf IAA and branch ZT. Additionally, the branch ABA/IAA ratio was positively correlated with the ABA/GA_3_ ratio.

At the JH site (Figure 5b), ZT levels in leaves and branches showed a significant positive correlation. The leaf ABA/GA_3_ ratio exhibited a highly significant negative correlation with leaf GA_3_ and a significant negative correlation with branch GA_3_, while being positively correlated with leaf IAA. The leaf ZT/GA_3_ ratio was significantly positively correlated with both leaf ZT and the leaf ABA/GA_3_ ratio and significantly negatively correlated with leaf GA_3_. A highly significant positive correlation was found between the leaf ABA/IAA ratio and branch ABA. The branch ABA/GA_3_ ratio was significantly positively correlated with leaf ABA and the branch ABA/IAA ratio and exhibited a highly significant positive correlation with branch ABA levels, while being significantly negatively correlated with branch GA_3_. The branch ZT/GA_3_ ratio showed significant or highly significant positive correlations with the leaf ABA/IAA ratio and the branch ABA/GA_3_ ratio. The branch ABA/IAA ratio exhibited significant or highly significant positive correlations with branch ABA, the ABA/GA_3_ ratio, and the leaf ABA/IAA ratio, while showing a significant negative correlation with branch IAA. Similarly, the branch ZT/IAA ratio was highly significantly negatively correlated with branch IAA.

In summary, the two sites exhibited distinct hormone regulation patterns. At the MH site, coordinated interactions between ABA and GA_3_ formed the core of the regulatory network, characterized by significant cross-organ correlations between leaves and branches. In contrast, hormonal regulation at the JH site was primarily governed by ABA-dominated ratios, with significant correlations largely confined within the branches. These findings suggest that *macadamia* trees exhibit site-specific hormonal response patterns, reflecting distinct regulatory adaptations to contrasting ecological environments.

## 3. Discussion

### 3.1. Relationship Between Endogenous Hormone Dynamics and Off-Season Flowering in Macadamia

Flowering in plants is a complex physiological process regulated by the interplay of genetic background, environmental conditions, and endogenous signals, among which plant hormones play a central role during key developmental stages such as floral induction, dormancy release, reproductive transition, and floral organ formation [18]. In this study, the temporal dynamics and potential regulatory roles of zeatin (ZT), gibberellic acid (GA_3_), indole-3-acetic acid (IAA), and abscisic acid (ABA) were systematically investigated based on twelve months of continuous monitoring at two sampling sites (MH and JH). Cytokinins, particularly ZT, are critical regulators of floral bud initiation and the transition from vegetative to reproductive growth in perennial woody species. These effects are primarily mediated through the promotion of cell division, modulation of source–sink relationships, and optimization of nutrient allocation, thereby facilitating reproductive switching [19,20,21]. In the present study, ZT levels in branches at both MH and JH showed a bimodal distribution from July to September, although the timing and magnitude of the peaks differed significantly between the two sites. At MH, ZT levels began to rise in July and peaked in September, closely corresponding with elevated ZT concentrations in leaves and the period of floral bud sprouting. These findings suggest that ZT may facilitate off-season floral induction by enhancing floral initiation signals and regulating carbon allocation. This observation implies that, even before seasonal cues have fully subsided, ZT-mediated floral initiation pathways are prematurely activated at MH, thereby triggering atypical reproductive development. In contrast, JH not only exhibited two major peaks of ZT but also showed a minor peak in March, coinciding with the expected seasonal floral initiation period. This suggests that ZT is involved not only in floral induction but also in subsequent stages such as floral organ development and anthesis [22,23]. Previous studies in perennial fruit trees, including apple and lychee, have demonstrated that a rapid increase in ZT prior to floral differentiation can activate key transcription factors and hormonal signaling pathways that enhance flowering potential [24]. Moreover, ZT operates within a synergistic regulatory network alongside IAA and gibberellins [25,26]. The advanced timing and sharpened peak of ZT signals observed at MH likely reflect region-specific ecological modulation of ZT biosynthesis, transport, and perception, ultimately contributing to off-season flowering.

Gibberellins (GA_3_) play a pivotal role in regulating flowering time and floral bud development. Moderate levels of GA_3_ promote floral induction, whereas excessive concentrations often suppress flowering, delay reproductive transition, and promote sustained vegetative growth [27]. In the present study, GA_3_ levels in MH leaves increased rapidly during the early stages, followed by a sharp decline and a prolonged period of low concentration. In contrast, GA_3_ concentrations at JH remained generally higher and more stable, with the onset of the low-GA_3_ phase occurring later than at MH. Previous studies have shown that GA_3_ can accelerate reproductive transition by upregulating flowering-related genes such as *SOC1* and *LFY* [28,29]. In *macadamia*, low GA_3_ levels have been reported to facilitate floral induction [30]. Therefore, site-specific differences in the timing of the low-GA_3_ threshold may be a critical factor contributing to off-season flowering.

Indole-3-acetic acid (IAA), a hormone essential for vegetative development, is typically associated with cell elongation and the differentiation of vegetative organs [31]. A progressive reduction in IAA concentration is believed to relieve its inhibitory effect on the reproductive transition, thereby promoting floral induction [32]. In this study, IAA levels in MH leaves exhibited pronounced troughs in March, May, and October, while branch IAA concentrations peaked in March. A similar pattern was observed at JH, with leaf IAA declining in March and October and a corresponding peak in branch IAA in March. This inverse trend between organs during the same period may reflect a regulatory mechanism governing IAA transport and spatial distribution during specific developmental windows [33]. Elevated IAA accumulation in branches during early floral induction may function as a localized signal that promotes floral meristem initiation or reprogramming of vegetative tissues [34]. Such spatiotemporal asynchrony in IAA distribution may play a crucial role in the hormonal regulation underlying off-season flowering. In comparison, IAA levels at JH remained relatively stable throughout the year, suggesting a more synchronized and consistent flowering rhythm.

Abscisic acid (ABA), in addition to its well-established role in abiotic stress responses, has also been implicated in floral transition through the regulation of downstream targets such as *FT* and *SOC1*. High ABA accumulation during floral bud differentiation has been shown to promote the formation of floral primordia [34,35]. In this study, peak ABA levels at the MH site occurred approximately two months earlier than those at JH, suggesting that early activation of ABA signaling may play a significant role in promoting off-season floral induction. Moreover, the timing of ABA peaks closely matched the observed off-season flowering period, indicating that ABA may function not only as a floral activator but also exert a biphasic regulatory effect, facilitating both the initiation and the sustained development of floral buds. In contrast, the delayed and attenuated ABA signaling observed at the JH site may represent an intrinsic physiological constraint that suppresses off-season flowering at this site. Notably, ABA levels at MH exhibited a distinct “up–down–up” trend between September and November, with peaks in September and November and a transient decline in October. This biphasic pattern may reflect a two-stage regulatory mechanism: an initial surge of ABA in September may facilitate floral induction, while a second rise in November could support floral organ development or enhance floral bud resilience under increasing abiotic stress (e.g., decreasing temperatures or water limitation) [36,37]. Conversely, the relatively flat ABA profile at JH suggests the absence of such dual-stage signaling, potentially contributing to the lack of off-season flowering at this site.

In summary, the occurrence of off-season precocious flowering in macadamia is not driven by a single hormone but rather results from the coordinated regulation of a multi-hormone signaling network comprising IAA, GA_3_, ZT, and ABA, acting in a temporally and spatially integrated manner. The pronounced ZT peaks, reductions in GA_3_ levels, periodic decline in IAA, and the earlier peak in ABA collectively constitute a hormonal profile conducive to disrupting conventional phenological rhythms and promoting the differentiation of off-season floral buds. These hormones act in a complementary fashion across different developmental stages, and their interaction—coupled with a compensatory mechanism involving multiple waves of floral bud differentiation—shapes the regional variations in flowering timing and rhythm in macadamia. This finding These findings provide critical insights into the endogenous regulatory basis of off-season flowering in this species.

### 3.2. Relationship Between Endogenous Hormone Balance Ratios in Macadamia and Off-Season Flowering

In plant development, while the absolute concentrations of individual hormones offer valuable physiological insights, the relative ratios among different hormone types often provide a more precise indication of signaling strength and the directionality of regulatory processes [38]. In this study, we examined the seasonal dynamics of key hormonal ratios, specifically ABA/GA_3_, ABA/IAA, ZT/GA_3_, and ZT/IAA, and their associations with off-season flowering. The results revealed that at the off-season flowering site (MH), these ratios in shoot tissues exhibited a marked increase from July to September, immediately preceding floral bud differentiation. This temporal pattern suggests the establishment of a hormonal milieu favorable to the initiation of reproductive development.

Further analysis revealed that the IAA/ABA and GA_3_/ABA ratios at the MH site displayed more pronounced temporal fluctuations, highlighting their potential importance in regulating the onset of off-season floral induction. Specifically, the IAA/ABA ratio peaked in August, closely aligning with the seasonal maximum of IAA. This pattern implies that the ratio may serve as a potential regulatory switch that facilitates dormancy release and activates pathways involved in floral differentiation. At the same time, the GA_3_/ABA ratio exhibited both an earlier rise and greater amplitude, suggesting a priming effect during the initial phase of floral bud induction and contributing to the physiological foundation for off-season flowering. Similar regulatory patterns have been reported in other fruit trees, such as apple (*Malus domestica*) and citrus (*Citrus* spp.), where a sharp increase in the IAA/ABA ratio prior to floral differentiation alleviates ABA-mediated repression and promotes the transition to reproductive growth [39,40].

The elevation of these hormone ratios not only reflects a shift toward promotive hormonal dominance but also demonstrates the plant’s ability to reconfigure its internal physiological state via the integration of hormonal regulatory networks. This ratio-based regulation enhances the temporal coordination and systemic alignment of hormonal signals, thereby strengthening the plant’s adaptive capacity to cope with environmental challenges such as seasonal mismatches and resource variability [41,42,43]. Accordingly, the intrinsic mechanism underlying off-season flowering in *macadamia* may involve the synergistic regulation of specific hormone ratios during key developmental periods. This mechanism offers an effective physiological compensation strategy when normal seasonal rhythms are disrupted.

### 3.3. Synergistic Effects of Endogenous Hormones in Macadamia and the Mechanism of Flowering Regulation

Multiple endogenous plant hormones interact through complex signaling crosstalk and integrated regulatory networks to coordinate the transition from vegetative to reproductive development [44]. In this study, correlation network analysis of hormone concentrations revealed significant positive associations among IAA, GA_3_, and ZT. This pattern suggests the presence of a synergistic hormone module that promotes flowering by jointly facilitating the activation of reproductive developmental pathways during floral bud induction. In contrast, ABA exhibited significant negative correlations with these promotive hormones and showed relatively low network connectivity, indicating its role as a limiting or inhibitory signal that constrains excessive activation within the hormonal regulatory system.

Comparative analysis of hormone interaction networks between sites further revealed that the off-season flowering site (MH) exhibited higher network density and stronger hormonal synergism. In this network, GA_3_ and IAA occupied central and highly connected positions, which underscores their pivotal roles in signal integration. As a key hormone promoting flowering, GA_3_ not only upregulates the expression of genes involved in reproductive development but also accelerates floral bud differentiation and organogenesis by activating essential floral regulators such as *LEAFY* and *SOC1* [18]. IAA likely contributes to floral morphogenesis by regulating cell division and elongation through signaling crosstalk with GA_3_ and synergistic interactions with ZT [45].

Although zeatin (ZT) did not exhibit substantial fluctuations in absolute concentration, its strong positive correlations with both indole-3-acetic acid (IAA) and gibberellic acid (GA_3_) suggest a functional role as a signal-modulating factor that contributes to the stability and robustness of the floral induction hormonal network [46]. Abscisic acid (ABA), a classical antagonistic hormone, maintained a consistently negative correlation pattern, supporting its role in fine-tuning floral induction by modulating the timing and amplitude of hormonal activation [47]. Collectively, these findings indicate a regulatory framework involving both synergistic and antagonistic interactions, wherein hormones achieve functional balance and signaling specificity through dynamic network coordination. The coordinated upregulation of IAA, GA_3_, and ZT not only counteracts ABA-mediated repression but also establishes an internal hormonal environment conducive to floral bud initiation. This integrated regulatory mechanism challenges the traditional model of single-hormone dominance and instead highlights the complexity and interconnectivity of hormonal network regulation in plants. These results are consistent with previously reported hormone interaction patterns in both model and crop species, including *Arabidopsis thaliana*, *Oryza sativa* (rice), and *Vitis vinifera* (grapevine) [48,49,50].

## 4. Materials and Methods

### 4.1. Site Description

Two field sites were selected for this study: an experimental site (MH) exhibiting off-season flowering and a control site (JH) with typical flowering phenology. The MH site is located in a commercial macadamia orchard in Nannuoshan Village, Menghai County, Xishuangbanna Dai Autonomous Prefecture, Yunnan Province (21°58′52.66″ N, 100°37′8.40″ E), at an elevation of approximately 1300 m. This plantation was established nine years ago with a tree spacing of 4.0 m × 6.0 m and is characterized by a uniform stand structure and vigorous tree growth. The JH site is located in the Macadamia Germplasm Resource Garden under the Ministry of Agriculture in Jinghong City (22°00′52.30″ N, 100°46′48.41″ E), at an elevation of approximately 550 m. This site functions as a conservation orchard and is managed under standardized horticultural protocols, ensuring consistently healthy tree development. Both sites lie within the northern tropical humid monsoon climate zone, characterized by warm, humid conditions with distinct wet and dry seasons. The rainy season generally extends from May to October. The MH site has a mean annual temperature of 21.5 °C and an average annual precipitation of approximately 1250 mm, whereas the JH site has a higher mean temperature of 24.3 °C and an annual precipitation ranging from 948 to 1515 mm.

### 4.2. Field Experiment Layout and Sample Collection

The field experiment was conducted in 2024 using macadamia nut trees (variety O.C.) of uniform age and growth status. Fifteen trees were randomly selected from each experimental plot, with one replicate assigned per three trees, resulting in five biological replicates per treatment. Sampling was conducted monthly on the 10th day between 08:00 and 10:00. From each tree, fully expanded second leaves from the apex of mature shoots and one-year-old branches were collected from the east, south, west, and north quadrants of the canopy. Each tissue type per replicate weighed no less than 15 g. Samples were immediately flash-frozen in liquid nitrogen and stored at −80 °C in an ultra-low temperature freezer. Endogenous hormones, including zeatin (ZT), gibberellic acid (GA_3_), indole-3-acetic acid (IAA), and abscisic acid (ABA), were quantified using high-performance liquid chromatography (HPLC). Total ZT content was measured without distinguishing between cis- and trans-isomers, as chromatographic separation of these isomers could not be achieved under the employed analytical conditions. Hormone identification and quantification were based on retention times and calibration curves established using commercially available standards.

### 4.3. Hormone Extraction and Quantification

#### 4.3.1. Instruments and Chemicals

Hormone quantification was carried out using a high-performance liquid chromatography (HPLC) system (LC-2030C, Shimadzu, Kyoto, Japan) equipped with a fixed-wavelength UV detector set at 210 nm. Additional laboratory equipment included a rotary evaporator(RE-52AA, Yarong Biochemical Instrument Factory, Shanghai, China), refrigerated centrifuge (Eppendorf 5810R, Eppendorf, Hamburg, Germany), ultrasonic homogenizer (Scientz-IID, Scientz, Ningbo, China), ultra-low temperature freezer (Thermo Scientific, Waltham, MA, USA), constant-temperature shaker (Zhicheng, Shanghai, China), pH meter (Mettler-Toledo, Zurich, Switzerland), fume hood (Labconco, Kansas City, MO, USA), and 0.45 μm microporous membrane filters (Millipore, Billerica, MA, USA). Standard compounds of zeatin (ZT), gibberellic acid (GA_3_), indole-3-acetic acid (IAA), and abscisic acid (ABA) were obtained from Solarbio Life Sciences (Beijing, China) and Macklin Biochemical (Shanghai, China), all with purities ≥ 96%. HPLC-grade methanol and glacial acetic acid were used as solvents. Other reagents included petroleum ether, ethyl acetate, citric acid (analytical grade), sodium diethyldithiocarbamate (used as an antioxidant), and cross-linked polyvinylpyrrolidone (PVPP, for phenolic compound removal), all purchased from Macklin Biochemical. Ultrapure water was used throughout the experiments.

#### 4.3.2. Sample Preparation Method

Exactly 3.0 g of finely chopped branch or leaf tissue was ground into a fine powder under liquid nitrogen. The powder was extracted with 30 mL of pre-chilled 80% methanol containing sodium diethyldithiocarbamate (30 mg·mL^−1^; adjusted based on sample weight). After 30 min of ultrasonic extraction in an ice bath, the samples were incubated overnight at 4 °C in darkness. The mixture was then centrifuged at 10,000 rpm for 20 min, and the supernatant was collected. The pellet was re-extracted with five volumes of 80% methanol, followed by ultrasonic extraction and a 4-h incubation at 4 °C in the dark. After centrifugation (6000 rpm, 10 min), supernatants were combined. To remove phenolic compounds, 0.1 g PVPP was added, thoroughly mixed, and shaken at 4 °C for 30 min, then centrifuged at 6000 rpm for 15 min. The supernatant was concentrated under reduced pressure at 40 °C until the methanol was completely removed. The aqueous residue was extracted three times with twice its volume of petroleum ether to remove pigments; the organic phase was discarded. The aqueous phase was acidified to pH 3.0 with 0.1 mol·L^−1^ citric acid and extracted three times with equal volumes of ethyl acetate. The combined ethyl acetate extracts were evaporated to dryness at 40 °C under reduced pressure. The residue was reconstituted in 1 mL of HPLC-grade methanol, filtered through a 0.45 μm membrane, and stored at 4 °C until analysis. All samples were analyzed in triplicate to ensure accuracy and reproducibility.

#### 4.3.3. Measurement Method

(1) Preparation of Standard Solutions

Standard compounds of zeatin (ZT), gibberellic acid (GA_3_), indole-3-acetic acid (IAA), and abscisic acid (ABA) were each accurately weighed at 5.00 mg and individually dissolved in HPLC-grade methanol. Each solution was then diluted to a final volume of 5.00 mL to prepare stock solutions at a concentration of 1.00 mg/mL. These stock solutions were stored at −20 °C in the dark. Subsequently, the stock solutions were mixed in appropriate ratios to prepare a combined standard mother solution, which was then serially diluted with HPLC-grade methanol to obtain a series of mixed standard solutions containing ZT, GA_3_, IAA, and ABA. All solutions were stored at 4 °C and remained stable for up to one month.

(2) Chromatographic Conditions

Chromatographic analysis was conducted on an InertSustain AQ-C18 column (4.6 × 250 mm, 5.0 μm; GL Sciences, Tokyo, Japan). The mobile phase consisted of methanol and 0.1% glacial acetic acid in water, delivered according to a gradient elution program detailed in Table 2. The column temperature was maintained at 30 °C, with a flow rate of 1.0 mL/min and an injection volume of 10 μL. Detection was carried out using a fixed-wavelength UV detector (Shimadzu, Kyoto, Japan) set at 210 nm. This wavelength was selected based on previous studies demonstrating sufficient UV absorbance and sensitivity for zeatin (ZT), gibberellic acid (GA_3_), indole-3-acetic acid (IAA), and abscisic acid (ABA) in plant tissue extracts, allowing for simultaneous detection without derivatization [51].

#### 4.3.4. Method Validation for HPLC Analysis

The HPLC method was validated according to established protocols for plant hormone quantification [52,53,54]. Calibration curves for zeatin (ZT), gibberellic acid (GA_3_), indole-3-acetic acid (IAA), and abscisic acid (ABA) exhibited excellent linearity, with correlation coefficients (R^2^) exceeding 0.996 (Table 3). The limits of detection (LOD) ranged from 0.01 to 0.05 μg/mL, while the limits of quantification (LOQ) ranged from 0.03 to 0.12 μg/mL. Recovery tests at low, medium, and high spiking levels yielded recovery rates between 85.3% and 95.7%, with relative standard deviations (RSDs) below 5%. Both intra-day and inter-day precision analyses showed RSDs below 3.5% for retention time and peak area, demonstrating high reproducibility and analytical reliability. The detection wavelength of 210 nm was selected based on previous studies that confirmed sufficient UV absorbance and sensitivity for the simultaneous detection of multiple phytohormones at this wavelength.

#### 4.3.5. Determination of Endogenous Hormone Content

The extracted solutions from flower or leaf tissues were injected and analyzed under the chromatographic conditions described above. The peak areas corresponding to each hormone were recorded and applied to the respective linear regression equations to calculate their concentrations. The endogenous hormone content in each sample was then determined using the following formula:

$$Endogenous\;hormone\;content(\mu g/g)=\frac{C\times V}{m}$$

where *C* is the concentration of the hormone in the sample (μg/mL), *V* is the final volume of the extract (mL), and *m* is the fresh weight of the tissue sample used for extraction (g).

### 4.4. Data Processing

Data were analyzed using one-way analysis of variance (ANOVA) in SPSS 27.0 to evaluate significant differences among treatments. Statistical analyses and visualization of endogenous hormone concentrations and their ratios were performed using R software (version 4.2.2). Pearson correlation coefficients were calculated using the cor()function, and correlation heatmaps were generated with the corrplot package to illustrate inter-hormonal relationships. For data not conforming to normality assumptions, Spearman’s rank correlation was calculated using the cor.test() function. Data reshaping and figure enhancement were conducted using the ggplot2 and reshape2 packages to ensure clarity and improve graphical presentation.

## 5. Conclusions

This study systematically elucidated the annual dynamics of key endogenous hormones and their coordinated regulatory mechanisms associated with off-season flowering in *Macadamia integrifolia* cultivated in high-altitude regions. Clear differences in phenological progression were observed between off-season and normally flowering sites, accompanied by region-specific and temporally variable patterns in both hormone concentrations and hormonal ratios. Compared with the normal-flowering site, floral bud differentiation at the off-season site occurred significantly earlier and was associated with persistently low GA_3_ levels as well as elevated concentrations of ZT and ABA. Furthermore, critical hormonal ratios, including ABA/GA_3_, ABA/IAA, and ZT/GA_3_, increased markedly during the early stages of floral induction. These changes suggest the establishment of an internal hormonal environment that disrupts conventional phenological rhythms and promotes the initiation of reproductive development. Collectively, these findings provide important theoretical insights into the endogenous regulatory networks underlying flowering phenology in *macadamia* and offer a scientific basis for the development of targeted, hormone-based strategies for flowering regulation. Such approaches hold considerable potential for enhancing environmental adaptability and stabilizing yield in high-altitude *macadamia* production systems.

## Figures and Tables

**Table 1:** Phenological survey of two macadamia planting sites.

Sampling Site	Initial Flowering Period	Peak Flowering Period	Fruit Enlargement Period	Fruit Oil Accumulation Period	Maturity and Harvest	Floral Bud Dormancy Break Period
MH	Oct–Nov	Jan	Feb–Apr	May–Jul	Aug	Aug–Sep
JH	Jan–Feb	Mar	Apr–May	Jun–Aug	Sep	Oct–Dec

Note: Month names are abbreviated in this table (e.g., Jan = January, Feb = February, Mar = March, etc.).

**Table 2:** HPLC gradient elution procedure of endogenous hormones in macadamia.

Time (min)	Acetonitrile (%)	Methanol (%)	0.1% Phosphoric Acid (%)
3	5	5	90
10	12	12	76
20	22	22	56
30	20	20	60
35	5	5	90
40	5	5	90

**Table 3:** Linear regression equations for endogenous hormone standards.

Component	Regression Equation	Correlation Coefficient	Linear Range (μg/mL)
ZT	y = 43,743x + 249,867	0.999	0.203–208.077
GA_3_	y = 9769.6x + 85,522	0.996	0.202–206.848
IAA	y = 75,525x + 17,728	0.999	0.202–206.848
ABA	y = 18,902x – 18,636	0.999	0.204–209.715

## Data Availability

Data will be made available on request.
